# Prevalence and family function associated with suicide-related behaviors in vocational college population of southern China: the mediating role of depression

**DOI:** 10.3389/fpsyt.2025.1523253

**Published:** 2025-02-20

**Authors:** He Wang, Yingyu Zhong, Yueyun Wang

**Affiliations:** ^1^ Department of Healthcare, Shenzhen Maternity and Child Healthcare Hospital, Southern Medical University, Shenzhen, Guangdong, China; ^2^ School of Public Health, Southern Medical University, Guangzhou, Guangdong, China

**Keywords:** suicide, family function, depression, young adults, South China

## Abstract

**Background:**

Suicide is positively associated with a range of psychological risk factors such as family function and depression. However, it remains unclear if depression mediates the association between family function and suicide in Chinese adolescents.

**Methods:**

We enrolled 14263 students from Shenzhen Polytechnic College, China. Family function, depression and suicide were assessed in these students by self-report measures, respectively. With path analysis and logistic regression, the mediating role of depression in the association between family function and suicide was analyzed.

**Results:**

In this study, the detection rate of suicide-related behaviors among vocational school students was 12.19%, among which the detection rates of suicide idea, suicide plan and suicide attempt were 11.59%, 4.26% and 2.70%, respectively. Depression played a significant mediating role in the influence of family function on suicide-related behaviors.

**Conclusions:**

Poor family function might increase the risk of suicide-related behaviors in vocational school students, and this effect was partly realized by affecting their mental health. Improving family function could not only improve adolescent’s psychological status, but also reduce their suicide-related behaviors.

## 1 Introduction

According to the latest data released by the World Health Organization (WHO), suicide has become the fourth leading cause of death among young people aged 15 to 29 worldwide in 2019, with more than 10,000 young people in China dying from suicide every year (1). Child suicide has become a serious public health problem (2). The occurrences of suicide idea, plan and attempt are key components in the formation of suicide, and the occurrence of any one or more types is considered to be suicide-related behaviors (3). According to the study, more than half of the young people who died by suicide had shown suicide-related behaviors before suicide (4). Suicidal behavior not only brings extremely serious harm to young people, but also has adverse effects on their families, peers, schools and society. Therefore, measures should be taken to intervene suicide-related behaviors among young people. In relation to this, family have been shown to play a critical role in the prevention of suicidal behavior during adolescence (5).

Family function has been defined as the interactions with and reactions to family members (6). First of all, many studies have analyzed the effects of family function on mental health and suicide-related behaviors in adolescents (5, 7). In the other hand, there has been evidence that adolescents who had self-harm experience suffered impaired family function (8). Family function, as an environmental factor, affects suicide-related behaviors more by influencing children’s psychological states (9). Research showed that lack of communication, family conflicts and academic pressure are important reasons for children’s suicide (2).

Previous study found that there was a correlation among family factors, depression and suicide (5). Further studies have discussed the mediating role of depression between family functioning and suicide-related behaviors among French and Spanish children (9, 10). The Three-Step Theory of Suicide suggests that pain and hopelessness are the causes of suicidal ideation, which are central symptoms of depression (11). Therefore, depression may play a mediating role between family function and suicide behaviors. However, the studies on the influence of family functioning on suicide-related behaviors among Chinese adolescents are still extremely limited (12). Previous research mainly regarded children who have attempted suicide-related behaviors as high-risk groups, and explored the influencing factors of suicide-related behaviors from three dimensions of individual, family and society, with a view to reducing such harm (13). In fact, understanding the effects of depression as a mediator would advance our comprehension of the mechanisms underlying the association between specific psychopathology and suicidal behavior (9), so as to provide targeted guidance for the prevention and intervention of young people’ suicide.

This study aims to describe the prevalence of suicidal behavior in a representative sample of Chinese young people in vocational school. This group represents a specific stage in the transition from adolescence to adulthood. Vocational school students often face rapid career preparation and employment pressures while still dealing with issues of personal identity and independence in early adulthood. This population may experience specific psychological stresses and challenges. Therefore, studying this group will not only help us better understand the developmental patterns of suicide-related behaviors, but also provide the basis for mental health interventions targeting this specific population. In addition, the aim of our study was to explore the possible preventive role of family function. Finally, we aimed to analyze the mediating role of depression in the relationship between family function on suicide-related behaviors.

## 2 Methods

### 2.1 Study population

This study was a cross-sectional study, which recruited the students in Shenzhen Polytechnic College from May to August in 2019. A total of 14263 subjects aged 15-30 years old were enrolled and finished the questionnaire. All questions in the questionnaire were set as mandatory. The students who were on leave or sick were excluded. Participants filled out an electronic questionnaire by scanning a QR code. All participants were asked to answer the questionnaires independently and to not discuss with others. After the questionnaire was collected, the questionnaires with obvious logic errors were excluded (Shenzhen vocational school has a requirement of 15 years old or older for admission, this study excluded those whose age were less than 15 years old). Finally, 12324 valid questionnaires were obtained. The effective response rate was 86.41%.

### 2.2 Assessment of family function, depression and suicide

Family function was measured by the Family APGAR Index (APGAR) (14) to understand the satisfaction of family member. It includes five dimensions: adaption, cooperation, growth, affection and intimacy. Each item was assigned with 0 ~ 2 points (almost never =0, sometimes =1, often =2). A score of 0 to 10 indicates good family functioning, a score of 4 to 6 indicates moderate family functioning impairment, and a score of 0 to 3 indicates severe family functioning impairment. In this study, the internal consistency of the questionnaire was good, and the Cronbach’s α coefficient of the scale was 0.890. The validity of the scale was also good (Kaiser-Meyer-Olkin coefficient =0.925, Bartlett’s sphericity test for significance *p*<0.001).

Depression were obtained by self-report in the questionnaire. The question about depression was a fill-in-the-blank question below: In the past 12 months, have you felt so sad or hopeless almost every day for 2 weeks or more that you stopped some of your daily activities?

Suicide-related behaviors assessment tools: Using the suicide-related behavior questions of Youth Risk Behavior Surveillance System (YRBSS), including suicidal ideation (have you ever seriously thought about attempting suicide in the last 12 months), suicide planning (do you have a specific plan for how you will attempt suicide in the last 12 months) and suicide attempts (how many times have you actually attempted suicide in the last 12 months). The questionnaire uses a scale of 1 to 4, where 1 means “never”, 2 means “happened once”, and 3 means “happened twice”. 4 indicates that it has occurred three or more times. In this study, the Kaiser-Meyer-Olkin coefficient = 0.642, Bartlett’s sphericity test for significance *p*<0.001, and Cronbach’s α was 0.706, which had a high reliability and validity.

### 2.3 Statistical analysis

Epidata 3.1 was used to input the questionnaire information. SPSS 26.0 and R 4.4.0 was used to analyze the data. The data cleaning method in statistics was used to filter out invalid values, and we check the consistency of data to ensure the validity and accuracy and reduce bias as much as possible. We conducted an *χ* ^2^ test to analyze the grade and household variables and a Student’s *t*-test for other variables. A Spearman’s correction was performed to analyze the relationships between family function, depression and suicide. Two-sided *p*-values less than 0.05 are considered to be statistically significant in this study.

Using AMOS 26.0, we performed path analysis to identify the relationships between family function, depression and suicide by structural equation modeling (SEM). The advantage of SEM is that it can simultaneously estimate multiple interdependent relationships, identify the potential variables of these relationships, and obtain the direct and indirect effects. Importantly, SEM can deal with multiple dependent variables simultaneously. Since each family function sub-score is highly correlated, depression, suicide and all family function sub-fractions are incorporated into one model by SEM to illustrate the correlation between each family function sub-fraction. SEM has been widely used in the research of psychology.

Mediation analysis was applied to further explore whether depression played a mediation role in the association of family function and suicide by using logistic regression and R mediation package 1.2.0. The test level is *α* = 0.05. In the mediation analysis, the relation between the independent variable family function and the dependent variable suicide was tested to see if it was significantly mediated by the variable depression. Path a. the association between family function and depression (the mediator). Path b. the association between family function, depression and suicide (the outcome). Path c. the multiplication effect of path a and path b.

## 3 Results

### 3.1 Socio-demographic features and clinical variables

The characteristics of the population enrolled were showed in **Table 1**. Totally, 12324 subjects were included in this study, with an average age of (19.74 ± 1.13) years. The detection rate of suicide-related behavior was 1502/12324 = 12.19%, and the detection rate of suicide idea, suicide plan, suicide attempt were 11.59%, 4.26% and 2.70%, respectively. Among the subjects, there were 5307 males (43.06%) and 7017 females (56.94%), and the detection rate of suicide idea behavior was higher in females, however, the detection rate of suicide attempt was lower in females. There were 6585 students (53.4%) in urban households and 5739 students (46.57%) in rural households. The detection rate of suicide-related behaviors in urban households was higher than that in rural households. The rates of suicidal idea, suicide plan and suicide attempt in depressed group were higher than those in non-depressed group (*P*<0.001). The five dimensions of family function and total scores of students with suicide-related behaviors were lower than those of students without suicide-related behaviors (*P*<0.001). The results were presented in **Table 2**.

**Table 1:** Incidence of suicide-related behaviors.

Variable	Suicide idea			Suicide plan			Suicide attempt		
	Yes (n=1429)	No (n=10895)	*P*	Yes (n=525)	No (n=11799)	*P*	Yes (n=333)	No (n=11991)	*P*
Gender			<0.001			0.475			0.004
Male	520 (9.8%)	4787 (90.2%)		234 (4.4%)	5073 (95.6%)		169 (3.2%)	5138 (96.8%)	
Female	909 (13.0%)	6108 (87.0%)		291 (4.1%)	6726 (95.9%)		164 (2.3%)	6853 (97.7%)	
Grade			<0.001			<0.001			<0.001
Grade 1	282 (12.8%)	1916 (87.2%)		111 (5.1%)	2087 (94.9%)		74 (3.4%)	2124 (96.6%)	
Grade 2	691 (12.0%)	5057 (88.0%)		265 (4.6%)	5483 (95.4%)		161 (2.8%)	5587 (97.2%)	
Grade 3	450 (10.3%)	3909 (89.7%)		145 (3.3%)	4214 (96.7%)		93 (2.1%)	4266 (97.9%)	
Grade 4	6 (31.6%)	13 (68.4%)		4 (21.1%)	15 (78.9%)		5 (26.3%)	14 (73.7%)	
Household			<0.001			0.001			0.019
Country	549 (9.6%)	5190 (90.4%)		206 (3.6%)	5533 (96.4%)		134 (2.3%)	5605 (97.7%)	
City	880 (13.4%)	5705 (86.6%)		319 (4.8%)	6266 (95.2%)		199 (3.0%)	6386 (97.0%)	
Depression			<0.001			<0.001			<0.001
Yes	557 (43.4%)	727 (56.6%)		254 (19.8%)	1030 (80.2%)		152 (11.8%)	1132 (88.2%)	
No	872 (7.9%)	10168 (92.1%)		271 (2.5%)	10769 (97.5%)		181 (1.6%)	10859 (98.4%)	

**Table 2:** Differences in the scores of the various dimensions of family function.

Variable	Suicide idea			Suicide plan			Suicide attempt		
	Yes	No	*P*	Yes	No	*P*	Yes	No	*P*
Family function									
Adaptation	0.96 ± 0.71	1.41 ± 0.66	<0.001	0.91 ± 0.74	1.38 ± 0.67	<0.001	1.03 ± 0.77	1.37 ± 0.68	<0.001
Cooperation	0.83 ± 0.74	1.32 ± 0.70	<0.001	0.82 ± 0.78	1.28 ± 0.72	<0.001	0.90 ± 0.81	1.27 ± 0.72	<0.001
Growth	1.05 ± 0.71	1.46 ± 0.64	<0.001	0.98 ± 0.75	1.43 ± 0.65	<0.001	1.04 ± 0.78	1.42 ± 0.65	<0.001
Affection	0.89 ± 0.73	1.38 ± 0.67	<0.001	0.88 ± 0.77	1.34 ± 0.69	<0.001	0.97 ± 0.80	1.33 ± 0.69	<0.001
Intimacy	1.06 ± 0.72	1.50 ± 0.62	<0.001	0.99 ± 0.75	1.47 ± 0.64	<0.001	1.01 ± 0.78	1.46 ± 0.64	<0.001
Total	4.79 ± 3.01	7.06 ± 2.88	<0.001	4.57 ± 3.21	6.90 ± 2.94	<0.001	4.95 ± 3.43	6.85 ± 2.96	<0.001

### 3.2 Common method deviation test

Since the questionnaire was filled out by class in this study, the results may be affected by common method bias, and Harman’s single-factor test was used for detection. The results showed that the eigenvalues of two factors was ≥ 1, and the first common factor could explain 45.44% of the total variation, which was less than 50% of the standard, so it could be inferred that there was no serious common method bias in this study.

### 3.3 Associations between family function, depression and suicide

The total and five sub-score of family function were negatively related with suicide-related behaviors and depression, and depression has a positive correlation with suicide-related behaviors. In the influence of family function on suicide-related behaviors, the correlation between affection with suicide idea was the strongest, with coefficients of -0.216, and the relation degree between resolve and suicide plan/suicide attempt were the highest, which were -0.137 and -0.100, respectively. The results were presented in **Table 3**.

**Table 3:** Correlation analysis of family function and suicide-related behaviors.

	1	2	3	4	5	6	7	8	9
2	0.515^**^								
3	0.396^**^	0.661^**^							
4	0.339^**^	0.262^**^	0.192^**^						
5	-0.232^**^	-0.144^**^	-0.093^**^	-0.153^**^					
6	-0.203^**^	-0.128^**^	-0.074^**^	-0.126^**^	0.862^**^				
7	-0.206^**^	-0.122^**^	-0.075^**^	-0.129^**^	0.899^**^	0.761^**^			
8	-0.189^**^	-0.127^**^	-0.085^**^	-0.132^**^	0.846^**^	0.668^**^	0.709^**^		
9	-0.216^**^	-0.124^**^	-0.076^**^	-0.147^**^	0.894^**^	0.692^**^	0.757^**^	0.694^**^	
10	-0.208^**^	-0.137^**^	-0.100^**^	-0.146^**^	0.855^**^	0.664^**^	0.700^**^	0.846^**^	0.781^**^

1. Suicide idea, 2. Suicide plan, 3. Suicide attempt, 4. Depression, 5. Family function, 6. Adaptation, 7. Partnership, 8. Growth, 9. Affection, 10. Resolve.

^**^ *P*<0.01.

### 3.4 Mediating effects and path analyses of depression

**Figure 1:**
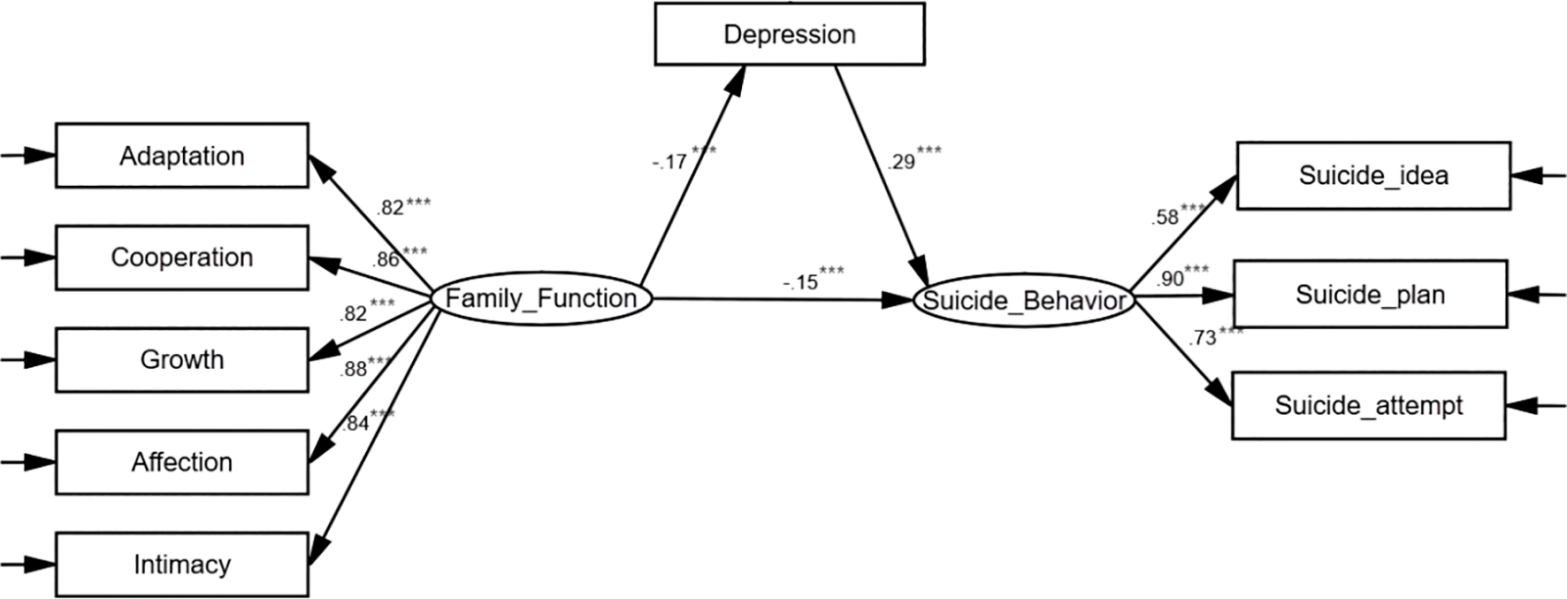
Model diagram of the mediating effect of depression on family function and suicide-related behaviors. The data in the figure are standardized coefficients, ^***^ *P*<0.001.

As the family function and depression were pair-wise correlated with suicide-related behavior, a model was established with family function as the independent variable, suicide-related behavior as the dependent variable, and depression as the mediating variable. Amos was used to estimate and test the parameters of the initial model (**Figure 1**). Model fitting indexes were as follows: RMSEA=0.078, NFI=0.968, IFI=0.968, TLI=0.954, CFI=0.968, indicating good fitting effect. Model explanatory power R^2^ = 0.34.

Family function can directly affect suicide-related behavior, and it can also affect suicide-related behavior through depression. The standardized coefficients of family function with suicide-related behavior, family function with depression, and depression with suicide-related behavior were -0.15 (*P*<0.001), -0.17 (*P*<0.001), and 0.29 (*P*<0.001), respectively.

Consistent with the above results according to the logistic regression analysis, family function was a protective factor for depression and suicide, *OR* (95% *CI*) = 0.842 (0.827, 0.858) and 0.817 (0.802, 0.833), respectively, and depression was a risk factor for suicide, *OR* (95% *CI*) =7.092 (6.199, 8.114). Z_a_ = -0.171/0.010 = 17.1, Z_b_ = 1.959/0.069 = 28.39, product distribution method was used to test the mediating effect of depression. The results showed that the 95% confidence interval of the mediating path “family function → depression → suicide related behavior” (Z_a_ × Z_b_) was (-0.366, -0.291). After adjusting for age and sex, Z_a_ = -0.171/0.010 = 17.1, Z_b_ = 1.972/0.069 = 28.58, and the 95% confidence interval of the mediating path “family function → depression → suicide related behavior” (Z_a_ × Z_b_) was (-0.363, -0.293), indicating that depression has a significant mediating effect on the relationship between family function and suicide, as shown in **Table 4**.

**Table 4:** Estimation of effects of family function on suicide mediated via depression.

Effect	Model 1				Model 2			
	*β*	*SE*	*P*	*OR* (95% *CI)*	*β*	*SE*	*P*	*OR* (95% *CI)*
Family function → depression	-0.171	0.010	<0.001	0.842 (0.827, 0.858)	-0.171	0.010	<0.001	0.842 (0.827, 0.858)
Family function, depression→suicide								
Family function → suicide	-0.202	0.010	<0.001	0.817 (0.802, 0.833)	-0.203	0.010	<0.001	0.816 (0.801, 0.832)
Depression → suicide	1.959	0.069	<0.001	7.092 (6.199, 8.114)	1.972	0.069	<0.001	7.182 (6.271, 8.224)

Model 1: crude.

Model 2: adjusted for age and sex.

## 4 Discussion

In this present study, we assessed the relationships between family function, depression and suicidal behaviors in urban areas of south China, and some interesting findings were suggested with different statistical analysis. In summary, we found family function was negatively associated with depression and suicidal behaviors, while depression showed a positive relationship with suicidal behaviors. Importantly, depression mediated the relationships between family function and suicidal behaviors.

### 4.1 Prevalence of suicide

The results of this study showed that prevalence of suicide-related behaviors was serious in vocational college population. The total detection rate was 12.19%, and the detection rate of suicide idea, suicide plan, suicide attempt were 11.59%, 4.26% and 2.70%, respectively, which were slightly lower than those of a meta-analysis on suicide idea (15.4%), suicide plan (6.4%) and suicide attempts (3.5%) among children and adolescents in English and Chinese publications from 2010-2020 (15). Another survey of middle and high school students in rural China showed that the detection rates of suicide ideation, suicide plan and suicide attempt were respectively 15.1%, 7.2% and 3.5% (16). One of the reasons for these differences may be regional differences, studies have shown that different provinces have different suicide rates, which was related to traditional culture and economic level (17). Our study was conducted in southern China with a large sample size, which can better represent the level of suicidal behavior among young people in Shenzhen area. The other reason may be differences in the definition of suicidal behavior, the target population and the timing of the survey (16). Compare with middle and high school students, vocational school students have less study pressure, which may be better for their mental health. Generally, our findings showed that Shenzhen vocational school students have a lower incidence of suicide-related behaviors compared to domestic and international suicide prevalence rates. Nevertheless, teenagers and young adults are in a period of psychological and physical upheaval, and we still need to take certain measures to reduce the incidence of suicide.

### 4.2 Family function and suicide

The other finding of our study was the total score of family function and five sub-dimensions (adaptation, partnership, growth, affection and resolve) had a significant predictive effect on suicidal behaviors, which was consistent with previous studies, suggesting that the young people’s suicidal behaviors has earlier causes, and worse family function may be a high risk factor. There are many influencing factors in the growth of children, but family has the greatest influence (18). Parental upbringing and family atmosphere play a vital role in their healthy growth. Studies by Harter et al. showed that children rely on families for universal support and develop healthy attachment patterns and emotional self-regulation (19). Family discord, lack of family communication and parent-child relationship tension are likely to lead to psychological problems in children (2). Existing studies have found that family factors including high family conflicts and low parental monitoring is significantly correlated with increased reports of suicidal ideation and suicide attempts (20), which is an important prerequisite of suicide. Sousa et al. conducted an integrated review of 29 literatures in 1980-2016, and their study suggested that stressful, depressing family atmosphere and lack of communication in family conflicts easily lead to suicide, and other factors such as parental separation or divorce, parental neglect and childhood abuse were risk factors for suicide-related behaviors as well (13). On the other hand, better family function can play a protective role in suicide-related behaviors. According to previous studies, family-centered therapy could reduce the frequency of suicidal ideation and suicide attempts (21). The underlying mechanism could be as follows. Firstly, when family function is maladaptive, young people might feel greater loneliness and reduce perception of family member’s feelings of care, need and love, thus increasing suicidal behavior (22). Secondly, according to the interpersonal theory of suicide, feeling disconnected from society and lacking family support can lead to a sense of belonging frustration, which is one of the main precursors of suicidal behavior in adolescence (23).

This study further found that family affection and intimacy had the highest negative correlation with suicide, that is, family affection and intimacy had a more protective effect on suicide. The reason might be that the stronger the emotional connection between family members, the more powerful the emotional support could be provided. When individuals encounter difficulties or psychological pressure, they could get comfort, understanding and help from family members (24). This close relationship of dependence and belonging makes individuals more likely to seek resources and support within the family when facing challenges, rather than commit suicide.

### 4.3 The mediating role of depression in the relationship between family function and suicide

The results of mediation effect analysis showed that depression played a mediating role in the influence of family function on suicide-related behaviors, which is similar to the results of previous research (9, 10). On the one hand, previous studies have shown that people with depressive disorder may have worse family function (25, 26), and research also suggested that family disfunction is a significant concomitant of depression and affected the course and clinical outcome of the disorder (27, 28). On the other hand, the association between depression and suicide has been extensive (29), and characterized by psychological, biological, and social mechanisms (30). In fact, with evidence that psychological problems are an important cause of suicide, among which depression is a major determinant of suicidal behavior, and the global rise in depression among adolescents has heightened concerns about suicide (31, 32). Therefore, depression plays an important role in the influence of family functioning on suicide-related behaviors.

From the above analysis, it could be seen that a supportive family environment is particularly important for young people’s mental health because it could serve as a psychological buffer against stress and challenges (33). In addition, educators could enhance student resilience by incorporating family engagement strategies and mental health education into school curricula, ensuring that students and families are aware of available mental health resources (34).

### 4.4 Strengths, limitations and conclusions

The strength of our study were as follows. Firstly, we performed a comprehensive analysis of the association between depression, family function and suicide-related behaviors, we conducted a variety of methods to assess the potential mediating role of depression in the associations between family function and suicide-related behaviors, which clearly illustrated the relationship between the three. Secondly, participants with chronic medical illness and mental disorders were not excluded in our study, therefore, the findings could be extended to special groups. Thirdly, one of the innovations in this study was to focus not only on a subset of adolescents, but also on young people, who were in the transition between adolescence and adulthood, and whose mental health was particularly important for the long-term health of society.

However, several limitations of our study should be noted. First of all, the current results were based on a cross-sectional study and cannot determine the causal relationships between family function and suicide-related behaviors. Therefore, this pathway should be validated in larger cohort studies. Secondly, although the sample size of our study was large, the participants were limited to vocational college students, which affected the universality of our results. Third, depressive symptoms were assessed using only a single question of initial screening rather than a depression scale. And the screening results could not diagnose depression. Therefore, more precise methods are needed to assess depression symptoms in the future so as to provide theoretical basis for early intervention of suicide.

In conclusion, this study provided important evidence that family function was negatively associated with suicide-related behaviors, and negatively related to depression in young Chinese adults. Depression has a positive correlation with suicide-related behaviors, and depression played an important role in the association between family function and suicide-related behaviors, which will provide new ideas and strategy for solving the problem suicide-related behaviors. It is worth mentioning that these findings need to be confirmed in future prospective studies that include more research factors (eg. anxiety and peer relationships/pressure etc.).

## Data Availability

The raw data supporting the conclusions of this article will be made available by the authors, without undue reservation.
